# Tumor-Infiltrating Lymphocyte Scoring in Neoadjuvant-Treated Breast Cancer

**DOI:** 10.3390/cancers16162895

**Published:** 2024-08-20

**Authors:** Noémie Thomas, Soizic Garaud, Mireille Langouo, Doïna Sofronii, Anaïs Boisson, Alexandre De Wind, Valérie Duwel, Ligia Craciun, Dennis Larsimont, Ahmad Awada, Karen Willard-Gallo

**Affiliations:** 1 Molecular Immunology Unit, Institut Jules Bordet, 1070 Brussels, Belgiumanais.boisson@hubruxelles.be (A.B.); 2 Anantomical Pathology Department, Institut Jules Bordet, 1070 Brussels, Belgium; 3 Anatomical Pathology Department, AZ Klina, 2930 Brasschaat, Belgium; valerie.duwel@klina.be; 4 Tumor Bank, Institut Jules Bordet, 1070 Brussels, Belgium; 5 Medical Oncology, Institut Jules Bordet, 1070 Brussels, Belgium

**Keywords:** breast cancer, neoadjuvant chemotherapy, tumor-infiltrating lymphocytes, immune biomarkers, digital pathology, lymphoid aggregates, tertiary lymphoid structures

## Abstract

**Simple Summary:**

TIL scoring has been recommended as a biomarker in routine clinical practice in breast cancer patients. Currently, the standard of care for early breast cancer is neoadjuvant treatment. Recent studies have demonstrated the additional predictive value of TIL scoring to the residual cancer burden after neoadjuvant treatment. Although guidelines have been published, the reliability of this biomarker in treated tumor samples has not yet been evaluated. Here, we show that there is good inter-pathologist reproducibility for TIL scoring in patients with clear residual tumor tissue. However, in patients with a (near-)complete response, there is not. This is significant because this demonstrates that it could be a reliable biomarker and help guide adjuvant treatment decisions.

**Abstract:**

Neoadjuvant chemotherapy (NAC) is now the standard of care for patients with locally advanced breast cancer (BC). TIL scoring is prognostic and adds predictive value to the residual cancer burden evaluation after NAC. However, NAC induces changes in the tumor, and the reliability of TIL scoring in post-NAC samples has not yet been studied. H&E- and dual CD3/CD20 chromogenic IHC-stained tissues were scored for stromal and intra-tumoral TIL by two experienced pathologists on pre- and post-treatment BC tissues. Digital TIL scoring was performed using the HALO^®^ image analysis software (version 2.2). In patients with residual disease, we show a good inter-pathologist correlation for stromal TIL on H&E-stained tissues (CCC value 0.73). A good correlation for scoring with both staining methods (CCC 0.81) and the digital TIL scoring (CCC 0.77) was also observed. Overall concordance for TIL scoring in patients with a complete response was however poor. This study reveals there is good reliability for TIL scoring in patients with detectable residual tumors after NAC treatment, which is comparable to the scoring of untreated breast cancer patients. Based on the good consistency observed with digital TIL scoring, the development of a validated algorithm in the future might be advantageous.

## 1. Introduction

Patients with locally advanced breast cancer (BC) are now routinely treated with neoadjuvant chemotherapy (NAC). This treatment helps to reduce tumor size and thereby facilitate breast conserving surgery. Importantly, the responsiveness of an individual patient to NAC provides important prognostic information [1] and is generally included in the pathology report. One frequently used approach is the Residual Cancer Burden (RCB) Calculator, which divides patients into four response categories linked with their prognosis: pathological complete response (pCR), RCBI, RCBII, and RCBIII, signifying increasing residual tumor burden [2]. A pCR signals a good prognosis and is used as a surrogate endpoint for long-term outcome in clinical trials, including those investigating immunotherapies [3]. Still, a small group of patients achieving pCR relapse, while approximately 50% of patients with an RCB III do not [4].

The tumor immune microenvironment, and more specifically tumor-infiltrating lymphocytes (TIL), have prognostic value for many different solid cancers, including BC [5]. The concept that a pre-existing immune infiltrate predicts better clinical outcomes was initially validated by scoring TIL in a large BC cohort [6]. Incremental increases in intra-tumoral or stromal TIL were found to be progressively associated with higher pCR rates after NAC. The ability to achieve a pCR was correlated with TIL as a continuous parameter, capped by patients with lymphocyte predominant BC (>60% intra-stromal TIL) who were those most likely to achieve a pCR. Subsequently, a study of BC patients enrolled in the BIG 02-98 adjuvant clinical trial revealed that patients with lymphocyte predominant BC, primarily the HER2+ and TN subtypes, have the best long-term clinical outcomes [7].

In the early TIL studies, many variations in scoring methodologies and cut-offs were used to evaluate the infiltrate, making dataset comparisons difficult. In 2013, the International Immuno-Oncology Biomarker Working Group was formed to address this issue, creating BC scoring guidelines in 2015 [8] and later updating and expanding them to include other solid tumors in 2017 [9,10]. TIL are typically scored on hematoxylin–eosin (H&E)-stained sections, a low-cost method readily available in most pathology labs worldwide. The effort of the TIL Working Group was to assist pathologists to achieve more standardized TIL scores. Ring studies to evaluate inter-pathologist reliability employing the guidelines found good agreement by an international consortium [11]. Further improvement was observed when additional tools, including tutorials and reference images, were provided to pathologists, all freely available on “https://www.tilsinbreastcancer.org/ (accessed on 12 August 2024)”. [12]. Good agreement on stromal TIL scoring in untreated BC was confirmed by two independent studies, including one from our laboratory [13,14]. A large, pooled analysis of six neoadjuvant [15] and nine adjuvant trials [16] confirmed the predictive value of TIL across all BC subtypes. Their association with survival in TNBC even reached level 1b evidence for clinical validity. Based on this body of work, in 2019, the St. Gallen International Consensus [17] and the ESMO guidelines [18] recommended routine TIL quantification as a continuous parameter for early BC.

Increasingly, data on various solid tumors have shown that organized TIL aggregates and, in particular, structured tertiary lymphoid structures (TLS) can orchestrate anti-tumor immune responses [19,20]. Interest in the characterization of TLS in different tumor types has been growing, particularly since the demonstration in 2022 of their importance in the response to immunotherapy [21]. In BC, studies demonstrated the predictive value of TLS in TNBC [22] and the prognostic importance of TLS in patients treated in the adjuvant setting [23,24]. In most studies, a multiple-marker immunohistochemistry (IHC) approach or TLS gene signatures are used, but there is no validated methodology [25]. Previous work from our group demonstrated that there is a good reliability for TLS scoring in untreated breast tissue on dual chromogenic (cIHC)-stained tissues [14].

TIL scoring in tumor resection tissues after NAC has been sporadically studied, suggesting there is added prognostic value for patients with a high residual burden. Patients with high or increased TIL following treatment were found to have better disease-free survival than those that did not [26,27,28]. The goal of the present study was to explore how to reproducibly score TIL in post-NAC surgical specimens. This can be challenging because chemotherapy induces variable morphological changes in the breast tumor tissue [29]. Recommendations for scoring TIL in this setting have been published, but their reliability has not been evaluated [30]. We examined the reliability of scoring TIL and aggregates/TLS in tissues from BC patients with various responses following NAC treatment. Using multiple blocks from the same patient, analyzing different regions of the tissue, employing various staining protocols, and implementing digital pathology allowed us to ascertain the best approaches for scoring TIL after NAC treatment.

## 2. Materials and Methods

### 2.1. Patient Samples

Formalin-fixed and paraffin-embedded (FFPE) tumor resection tissues were collected from 68 out of 75 selected patients who received standard-of-care NAC (taxanes and anthracyclines) prior to surgery. Eight patients were excluded from this study due to poor quality FFPE tissue or absence of residual tumor tissue (Figure S1).

Patients were divided into two groups based on their response to treatment. The Residual Disease Cohort (*n* = 33) included patients with residual tumor nests at the primary tumor site. The Good Response Cohort included patients who achieved or nearly achieved a pathological complete response (*n* = 27) at the primary tumor site. Near-PCR patients with minimal remaining tumor cells were included in this cohort based on the resemblance of the scored TIL area to that of patients achieving pCR. The cut-off used was 5% tumor cellularity because of the limited sensitivity for identification of residual tumor areas below this cut-off. The clinicopathological characteristics for patients in both cohorts are summarized in Table S1. Two of the patients initially diagnosed with pCR by routine examination were found to contain substantial residual tumors by our study pathologists and thus were included in the Residual Disease Cohort. Patients treated at the Institut Jules Bordet who signed their informed consent were included in this study, which was approved by the institutional IJB’s ethics committee (CE-1981).

### 2.2. Histological Staining

FFPE tissue sections (4 µm) were cut from blocks pre-selected by pathologists routine reading of H&E-stained tissue sections. Freshly cut tissue slides were preheated at 56 °C for 4 h to reduce tissue detachment during chromogenic immunohistochemistry (cIHC) staining. Dual cIHC was performed using a Ventana Benchmark XT with our optimized protocol for CD3 plus CD20 to stain T cells and B cells, respectively [31].

### 2.3. Pathological Assessment

**Figure 1:**
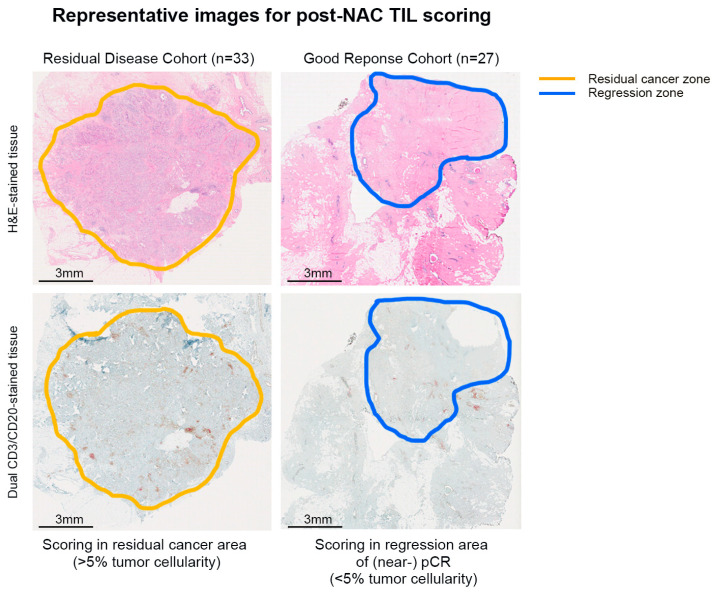
Representative images of the scoring areas defined by the pathologists are shown.

TIL scoring was independently performed by two experienced pathologists (RdW, VD) on both H&E- and dual CD3/CD20 cIHC-stained post-NAC surgical tissues. The pathologists defined two scoring areas: (1) the residual tumor area used to calculate RCB class and (2) a larger area that included the regression zone. The Good Response Cohort was only scored in the regression area, defined as the area of “young” fibrotic scar tissue where tumor cells resided before NAC (Figure 1). When the anatomo-pathological report described tumors with a diameter >20 mm, multiple blocs (one for every 10 mm) were scored, with the block containing the largest and most representative tissue area chosen for the final analysis.

Intra-tumoral and stromal TIL, defined as the percentage of the area occupied by mononuclear inflammatory cells in each region [8], were scored on H&E-stained tissues. T and B cell TIL were separately scored on dual CD3/CD20 cIHC-stained tissues [31]. Pathologists scored TIL as a continuous variable following the examples and guidelines from the TIL Working Group on the website [12]. Regions with necrosis, adipose tissue, normal ducts, carcinoma in situ, and crush artifacts were excluded. Aggregates were defined as areas of clustered TIL (H&E) or clusters of random, unorganized T and B cell TIL (CD3/CD20 cIHC). TLS were defined as B cell TIL clustered in a dense follicle surrounded or adjacent to a T cell TIL area (CD3/CD20 cIHC; examples shown in Figure S2). All aggregates and TLS were scored irrespective of their location relative to the residual cancer zone.

Stromal TIL scores from 55 matched biopsies were used as an internal control (Figure S3). The inter-pathologist concordance for stromal TIL compared favorably with our previous study on untreated breast cancer [14].

### 2.4. Digital Scoring

Dual CD3/CD20 cIHC-stained tissues from the Residual Disease Cohort were scanned and digitally scored using the HALO^®^ image analysis platform (version 2.2, Indica Labs, Albuquerque, NM, USA). The residual cancer area was first identified by a pathologist and manually annotated (Figure S4A). The software was first trained using regions of interest from various breast cancer samples with a diversity of morphological features and treatment responses using deep machine learning to classify tissue zones using the learning by example classifier (Figure S4B). The residual tumor was divided into three different categories: tumor, stroma, and disregarded tissue (corresponding to excluded areas according to the Working Group guidelines). Next, using the Multiplex IHC module, cellular segmentation was trained and optimized. Third, immunophenotyping for CD3+ cells (brown), CD20+ cells (red), and other cells (bleu nucleus) was performed based on respective staining thresholds. The optimized protocol was saved and applied to the annotated residual cancer areas of all the samples. T and B cell TIL were scored separately, with their individual scores used to calculate the total TIL score.

### 2.5. Statistical Analysis

Logarithmic transformation of the TIL scores was used to approximate normal distribution. A value of 1.055 was added to each score to avoid missing values after log-transformation of the scores with an original value of zero. The agreement of TIL scores was evaluated using Bland–Altman comparison plots [32]. Ratios were plotted against the geometric mean of each sample; the average ratio (central blue line on the plots) was calculated and plotted with the 95% agreement interval (wide blue lines), where ratios are expected to fall when normally distributed. Additionally, the correlation of TIL scores was analyzed using Passing–Bablock regression analysis [33]. The intercept and slope measure the constant and proportional bias between the two methods, respectively. The reproducibility index for these scoring methods was calculated using the CCC with a 95% confidence interval and is presented as a forest plot [34]. The CCC considers both the deviation from the best fit line and the 45° line. The interpretation of the scores is reported in comparison to results for inter- and intra-pathologist reproducibility in untreated breast cancer [11,14]. Analyses were performed using GraphPad Prism version 8.0.1 (GraphPad Software, Boston, MA, USA, “www.graphpad.com (accessed on 12 August 2024)” and R Statistical Software (v4.0.2; R Core Team 2020) and the packages epiR (R package version 1.0-14) [35] and MethComp (R package version 1.30.0) [36].

## 3. Results

### 3.1. Inter-Pathologist Concordance for Scoring TIL

**Figure 2:**
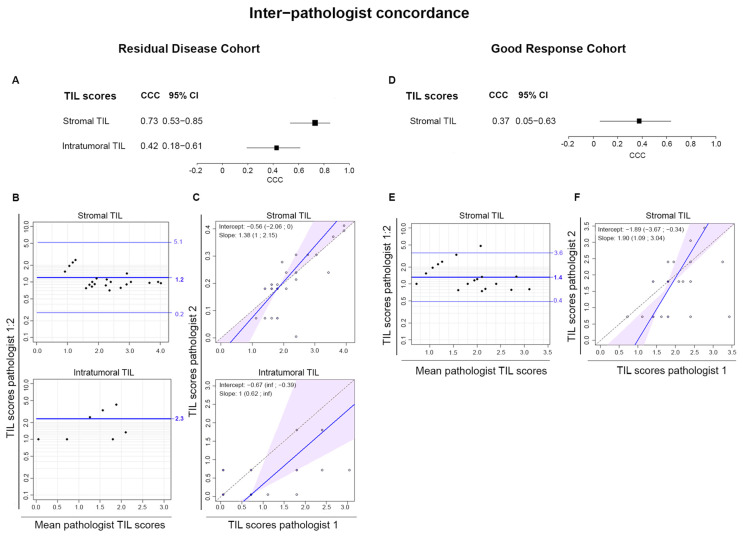
Scores from pathologists 1 and 2 were compared for the H&E-stained tissues. (**A**,**D**) The forest plot shows the concordance correlation coefficient (CCC) with the 95% confidence interval (CI). (**B**,**E**) Bland–Altman plots for stromal and intra-tumoral TIL scores are shown as a ratio of pathologist 1 to pathologist 2 (*y*-axis) plotted versus the mean score for each sample (*x*-axis). The mean ratio (central line) of these scores with the 95% limits of agreement are shown as horizontal lines. (**C**,**F**) Passing–Bablock regression analysis for stromal and intra-tumoral TIL scores from pathologist 1 (*y*-axis) compared to pathologist 2 (*x*-axis). The regression lines with the 95% CI (colored band) are drawn. Constant and proportional bias are indicated by the intercept and slope of the regression line, respectively.

The tumor tissues removed at surgery from the Residual Disease Cohort (Figure 1) were scored for stromal and intra-tumoral TIL by two pathologists on H&E- and dual CD3/CD20 cIHC-stained tissues. The CCC value revealed good agreement between pathologists for scoring stromal TIL on H&E-stained tissues (0.73 with 0.53–0.85 CI) (Figure 2A). In the Bland–Altman plots, most TIL score ratios are close to one, confirming these data (Figure 2B). The subtle differences are likely due to the different statistical analyses applied for CCC and Bland–Altman, which makes the latter more sensitive to deviating values. Using Passing–Bablock regression analysis, where the regression line for stromal TIL approximates a perfect correlation (Figure 2C), concordance was poor for scoring intra-tumoral TIL, reflected by the CCC and in the graphs. Our pathologists identified different reasons for the outliers: (1) in the Good Response Cohort, it is often difficult to identify all the remaining tumor cells and scoring area; (2) in residual tumors with low TIL scores, the visual difference between 5 and 10% is only small but can have a big effect on statistical analysis; and (3) in some residual tumors with higher TIL infiltration, this can be very heterogeneous, making it difficult to calculate a mean score.

Stromal TIL (but not intra-tumoral TIL; see Section 2) were also scored in the regression area of tissue from the Good Response Cohort. These scores revealed poor inter-pathologist concordance with a CCC value of 0.37 (Figure 2D), supported by the Bland–Altman and Passing–Bablock plots (Figure 2E,F). TIL scores in CD3/CD20-stained tissues gave similar results (Figure S5), although these tissues have a slightly better concordance for intra-tumoral TIL. Overall, these data demonstrate consistency for scoring stromal, but not intra-tumoral, TIL in the Residual Disease Cohort. Concordance in the Good Response Cohort was inferior, warranting caution when interpreting TIL scores from this patient group.

### 3.2. Comparison of Scoring TIL on H&E- versus CD3/CD20-Stained Tissues

**Figure 3:**
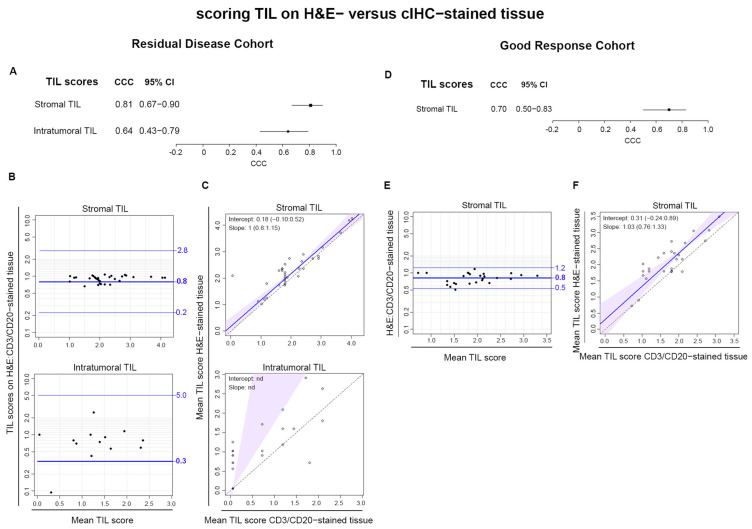
Pathologist TIL scores on H&E- versus CD3/CD20-stained tissue were compared (**A**,**D**). The forest plot shows the concordance correlation coefficient (CCC) for H&E- versus CD3/CD20-stained tissue (**B**,**E**). Bland–Altman plots demonstrate the ratio of TIL scores on H&E- to CD3/CD20-stained tissue (*y*-axis) plotted against the geometric mean scores for each sample (*x*-axis). (**C**,**F**) Passing–Bablock regression analysis for TIL scores on H&E- (*y*-axis) compared to CD3/CD20-stained tissue (*x*-axis).

Comparative analysis of the geometric mean scores for stromal and intra-tumoral TIL from H&E- and CD3/CD20-stained tissues revealed excellent concordance for stromal TIL in the Residual Disease Cohort, with a CCC of 0.81 (CI 0.67–0.90) (Figure 3A). This is maintained by the Bland–Altman plots (Figure 3B) and almost perfect Passing–Bablock regression analysis (Figure 3C). The intra-tumoral TIL scores were again poorly correlated with the differentially stained tissues, and this is confirmed by the inability to calculate a regression line. The mean ratios for the Bland–Altman plots and the intercepts of the regressions (<1 and >0, respectively) suggest a constant bias towards higher scores on CD3/CD20-stained tissues. Lower concordance was observed for stromal TIL scores from the Good Response Cohort with a CCC of 0.7 (Figure 3D). This is reinforced by the larger deviation from complete concordance in the Bland–Altman plot (Figure 3E) and Passing–Bablock regression analysis (Figure 3F). These data show there is an overall good agreement for TIL scores on these alternate stained tissues, again better for stromal TIL and the Residual Disease Cohort.

### 3.3. Comparison of TIL Aggregate Scores

The number of TIL aggregates were independently scored on H&E- and CD3/CD20-stained tissues (representative images in Figure S2; note: patients had low to intermediate numbers of aggregates and TLS were found in only two tumors, thus making analysis of the latter impossible). Overall, there was good inter-pathologist agreement for aggregate scores on H&E-stained tissues from the Residual Disease Cohort and H&E- and CD3/CD20-stained tissues from the Good Response Cohort (Figure S6). The Bland–Altman plots show divergent ratios for some tumors, reflecting important differences in aggregate scores between our pathologists. A comparison of mean pathologist scores between H&E- and CD3/CD20-stained tissues revealed moderate agreement for both cohorts (Figure S7). The Bland–Altman and Passing–Bablock analyses again show that some are highly divergent. While these data demonstrate a good overall consistency for scoring aggregates in both cohorts, which is better on H&E-stained tissues, tumors with wide variation in aggregate scores require further concentration.

### 3.4. Scoring TIL in Heterogeneous Residual Tumor Tissue

**Figure 4:**
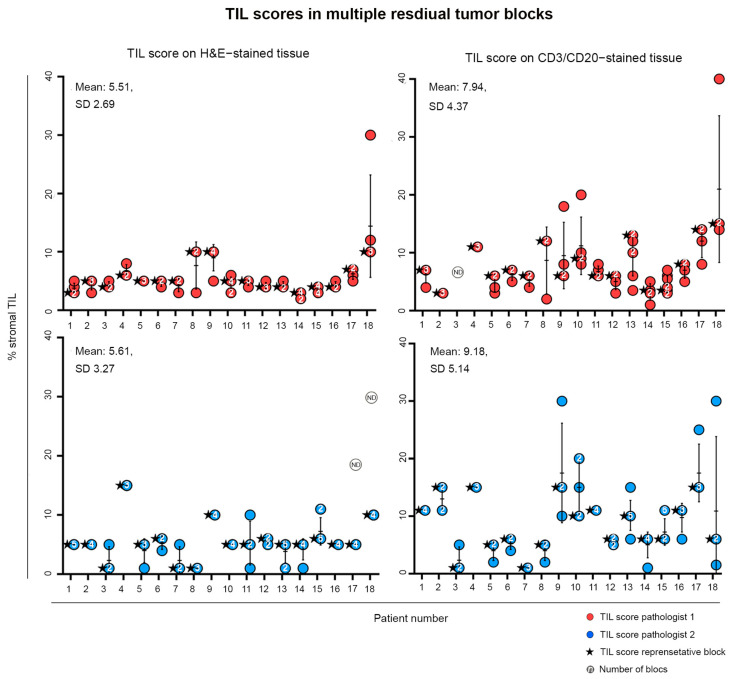
TIL scores on H&E- and CD3/CD20-stained tissue for all blocks are shown per patient. If multiple blocks had the same TIL score, the number of blocks with that score is indicated in the dot for the corresponding score. The score of the selected block used for the comparative analysis is indicated by a black star. The mean score is indicated by a black line.

Multiple blocks were collected and scored from patients with residual tumors >20 mm (*n* = 18; Residual Disease Cohort). Intra-patient heterogeneity in post-NAC TIL scoring was examined by plotting stromal TIL scores from all available blocks for each individual patient (Figure 4; 3–8 blocks/patient). Minor variation was detected in stromal TIL scores between intra-patient blocks, with scores for the CD3/CD20-stained tissues more variable, standard deviation 4.37 versus 2.59 for H&E-stained blocks. In general, this was due to one outlier block. Stromal TIL scores for the chosen representative block were comparable to scores from most of the other blocks of the same patient.

Retrospective analyses revealed that the few divergent blocks were characterized by relatively small tumor cell areas. A good correlation was observed between TIL scores from the residual cancer area and the residual cancer plus the regression area. Scores for the latter were similar or slightly lower (Figure S8). These data suggest that scoring TILs on one carefully selected block reflects the entire residual tumor but should not include the regression area for tumors with residual disease.

### 3.5. Digital Pathology

**Figure 5:**
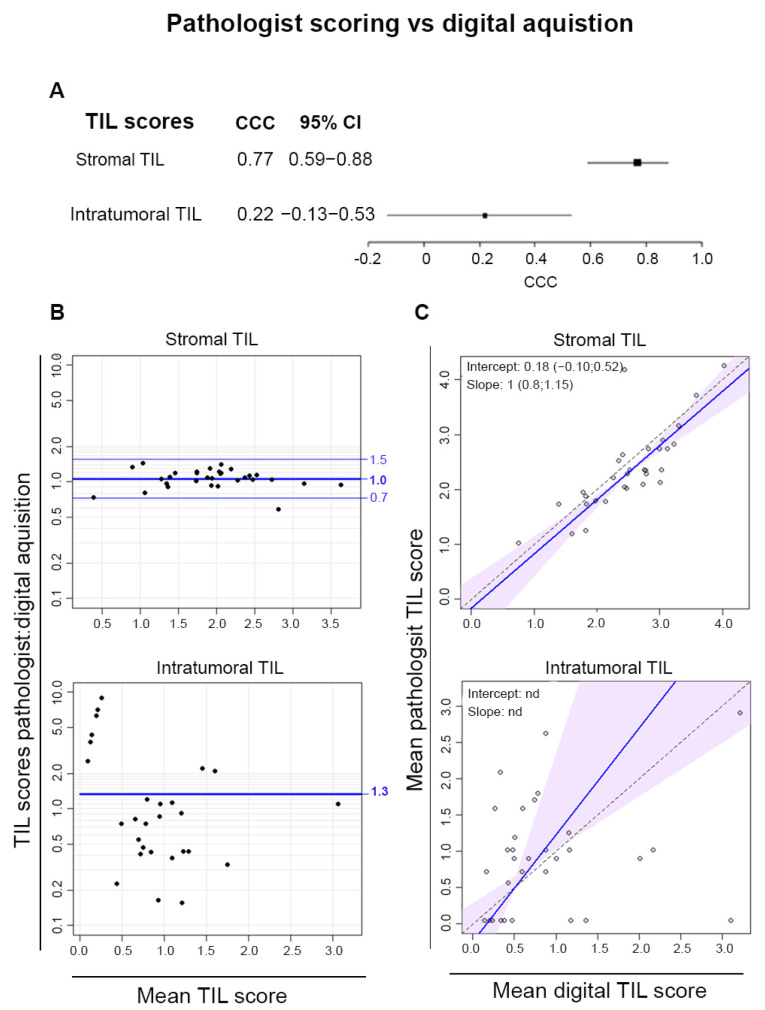
TIL scores obtained by digital analysis were compared to the mean pathologist TIL score from CD3/C20-stained tissue. (**A**) A forest plot shows the CCC values. (**B**) Bland Altmann plots demonstrate the ratio of mean pathologist scores to digital scores (*y*-axis) plotted versus the mean score for each sample (*x*-axis). (**C**) Passing–Bablock regression analysis shows mean pathologist scores (*y*-axis) compared to digital scores (*x*-axis).

Digital pathology scoring was compared to the mean pathologist scores for the CD3/CD20-stained tissues in the Residual Disease Cohort. There was a good agreement between the stromal TIL scores, reflected by CCC values of 0.77 and mean ratios close to one in the Bland–Altman plot (Figure 5A,B). The regression line shows an almost perfect correlation for stromal TIL scores on the Passing–Bablock regression plots (Figure 5C). In contrast, agreement between scoring by pathologists and digital pathology for intra-tumoral TIL was poor. Thus, when restricted to the stroma, where TIL are at higher concentrations, these data demonstrate that digital scoring is well correlated with pathologists’ scoring of TIL.

## 4. Discussion

Over the last decade, our understanding about the value of immune cell infiltration in solid tumors, including BC, has grown rapidly in parallel with the advent of cancer immunotherapy and increases in the frequency of treatment in the neoadjuvant setting. Large, pooled analyses of multiple neo-adjuvant trials have demonstrated the clinical value of scoring TIL in BC, leading to the recommendation for routine TIL quantification in early BC patients [18]. It is clearly important to the clinical decision-making process today that accurate quantification of immune activities in individual tumors becomes standard. This will necessitate having a reliable and reproducible methodology for quantification of the tumor immune microenvironment.

TIL scoring in post-NAC samples in some early studies yielded contradictory results. A pooled analysis of all BC subtypes found an association between high stromal TIL and worse clinical outcomes [37,38]. Studies centered on the HER2+ and TN subtypes revealed that both high stromal and intra-tumoral TIL signaled a better prognosis, particularly when there was a high residual tumor burden [39,40,41]. Asano et al. [42] showed that adding TIL to the RCB categories (named “RCB-TILs” with TIL scored as either positive or negative) was a better prognostic indicator than either alone across all BC subtypes. These findings were obtained using the TIL scoring methodology described for untreated BC. However, a small ring study found only modest inter-pathologist concordance (ICC 0.59) in post-treatment TNBC samples, emphasizing that these data need to be interpreted with caution when using approaches designed for untreated tumor samples [30]. Due to these constraints, specific recommendations for TIL scoring in the post-treatment setting were published as a guide to improve TIL scoring reliability post-NAC.

Employing the published recommendations, the present study evaluated the reproducibility of post-NAC TIL scoring, finding there was a good inter-pathologist agreement when scoring stromal TIL on surgical specimens with residual disease. This parallels TIL scoring results for primary tumors, where stromal TIL were more reproducibly scored between pathologists [11,14]. The reasons for outlier values in this study originate from the specific difficulties of scoring in treated tumor samples. When TIL scoring is performed using carefully selected blocks containing large residual tumor areas, the results are representative of the whole tumor. Pathologists trained in both post-NAC tumor evaluation and TIL scoring are instrumental for using TIL in routine clinical practice [43,44].

The slightly lower concordance observed in comparison with studies of primary, untreated BC is likely due to several factors. First, a standardized methodology for identifying residual tumor tissue following treatment has not yet been established. The updated TIL scoring recommendations suggest using the area defined as the tumor bed for the RCB Calculator [2]. At the moment, this is not implemented in all routine pathology laboratories. Inter-pathologist concordance for RCB scores is overall good, but tumor bed size estimations remain an important discordant factor [45]. Additionally, in tissues with scattered tumor foci within a large scar zone, the extent of the area to score is still being questioned [43]. Our hypothesis was that the stromal area directly surrounding the tumor cells is likely most important, which led the pathologists to score accordingly. This view is supported by TIL scoring data from larger areas inclusive of the regression zone, which generally have lower scores lacking clinical relevance. The poorly defined boundary for scoring could, however, be partly responsible for the reduced concordance compared with untreated BC. In the Good Response Cohort with <5% tumor cellularity, there was more difficulty in identifying the scoring area. The regression zone, characterized by scar tissue where the tumor was previously located, had no clearly defined boundaries, and thus there was only moderate concordance for scoring TIL in this area. In studies that include pCR patients for research purposes (some referred to above), TIL scores should be interpreted with caution. Comparable to findings in untreated breast cancer samples, concordance for intra-tumoral TIL scoring on H&E- and cIHC-stained tissues is poor, and these scores therefore cannot reliably be used in clinical or research settings.

We and others have previously demonstrated the utility of digital TIL scoring in primary BC patients [14,46,47]. More performant deep learning neural networks have enabled the development of computational TIL assessment algorithms for H&E-stained slides, where this was previously based on IHC-stained cell markers [48,49]. In colorectal cancer, an AI-assisted immune assay was even proven superior to pathologist scoring for predicting relapse [50]. There are numerous limitations that make digital TIL scoring unsuitable for routine clinical pathology today, but efforts to introduce artificial intelligence into digital pathology suggest that this will likely be practicable in the future [51,52]. Whether the same algorithms will be applicable in neoadjuvant-treated tumor samples remains to be seen, but Yoneyama et al. developed a super-TIL score that correlates well to manual TIL scoring in pre- and post-radiation BC [53]. In this study, we found a good concordance between digital pathology and mean pathologist scores for the CD3/CD20 cIHC-stained tissues, and although these stains may require additional handling and slightly higher cost they do afford some clear advantages, including: (1) the two major TIL phenotypes can be simultaneously and quickly quantified [54]; (2) TIL subpopulation organization into functional structures, such as TLS, can be scored [14,55]; (3) in the post-NAC setting, digital approaches to score TIL parameters that are currently unfeasible for pathologists, such as scoring stromal TIL within a well-defined perimeter of remaining tumor cells [56]; and (4) staining for a tumor cell marker to improve the segmentation of intra-tumoral versus stromal areas in an effort to improve intra-tumoral TIL scoring, similar to what was performed for Ki67 [57]. Ultimately, an algorithm combining automated RCB scoring with TIL scoring and distribution provides pathologists with a powerful tool to use in routine clinical practice.

A limitation of our study was the small number of patients that were then divided into two groups based on treatment response. This patient cohort also included a limited number of tumors with TIL aggregates and very few TLS further complicating the statistical analysis. However, even though the pathologists were unfamiliar with scoring aggregates, there was good agreement for most tumors in both groups. These data need to be confirmed on a larger patient cohort from a multicenter trial with scoring by a larger group of pathologists. While we showed that digital TIL scoring was correlated with pathologist scores, work is still needed to generate a reliable algorithm that can be used for routine clinical pathology. Whether similar findings apply to TLS requires a comparable evaluation using a cohort with higher frequencies of these structures, perhaps selected on baseline TIL. Currently, most studies have demonstrated the prognostic importance of TLS in cancer [58], making this an important future direction.

## 5. Conclusions

The present study confirms that scoring stromal TIL in residual tumor post-NAC treatment can be a reliable biomarker by trained pathologists and when the updated guidelines are respected. Open access to training images for TIL scoring in post-treatment samples via a website could also help improve concordance between pathologists, as it did for untreated tumors. Currently, clinical trials demonstrating the prognostic value of TIL scores in post-NAC residual tumors demonstrate conflicting results. However, scoring TIL next to the residual cancer burden can add prognostic value in patients with substantial residual tissue. This can support clinicians in the decision for the need of adjuvant treatment for patients with residual cancer post-NAC. New clinical trials using the updated TIL scoring guideline for post-treatment samples should be created so TIL scoring can be implemented as a biomarker in routine clinical practice to aid adjuvant treatment decision-making.

## Data Availability

All data used in this study can be made available upon reasonable request.

## Supplementary Materials

The following supporting information can be downloaded at: https://www.mdpi.com/article/10.3390/cancers16162895/s1. Table S1: Summary of the clinicopathological data; Figure S1: Flow chart of sample collection and division into the two cohorts; Figure S2: Representative Images of TIL aggregates and TLS; Figure S3: Inter–pathologist concordance in untreated biopsies; Figure S4: Example of digital TIL quantification with the HALO^®^ image analysis platform; Figure S5: Inter–pathologist concordance in CD3/CD20-stained tissue; Figure S6: Inter–pathologist concordance in for aggregate scores; Figure S7: Comparison of aggregate scores from H&E and CD3/CD20-stained tissue; Figure S8: Comparison of Stromal TIL scores from the RCB and RCB + regression area.
