# Supplementary material for: Tumor-Infiltrating Lymphocyte Scoring in Neoadjuvant-Treated Breast Cancer
Source: Cancers (Basel). 2024 Aug 20;16(16):2895. doi: 10.3390/cancers16162895 (PMC11352458; doi:10.3390/cancers16162895)
Supplement: Supplementary file 1 [file cancers-16-02895-s001.zip › Supplementary data.pdf]

Supplementary Materials

Table S1. Summary of the clinicopathological data of patients included in the two cohorts.

Supplementary figures

Supplementary figure S1. Flow chart of sample collection and division into the two cohorts

Supplementary figure S2. Representative Images of TIL aggregates on H&E- and CD3/CD20-stained tissue. On H&E-stained tissues, TIL aggregates could not be further characterized. On CD3/CD20-stained tissue, TIL aggregates with organization in a central B cell follicle and a margin of T cells were identified as a tertiary lymphoid structures (TLS).

Supplementary figure S3. Stromal TIL scores on H&E- and CD3/CD20-stained tissue were compared on untreated tumor biopsies. **A.** The forest plot shows the concordance correlation coefficient (CCC) with the 95% confidence interval (CI) for inter-pathologist agreement on stromal TIL scoring. **B.** Bland-Altman plots for stromal TIL scores shows the ratio of pathologist 1 to pathologist 2 (y axis) plotted against the geometric mean score for each sample (x axis). The mean ratio (central line) of these scores with the 95% limits of agreement are shown as horizontal lines. **C.** Bland-Altman plot for stromal TIL scores show the ratio of scores on H&E- to CD3/CD20-stained tissues (y axis) plotted against the geometric mean score (x axis). The mean ratio (central line) with the 95% limits of agreement is shown as a horizontal line. Passing-Bablock regression plot of stromal TIL score on H&E- versus CD3/CD20-stained tissues. The regression line with the 95% CI is indicated as a colored band. The constant and proportional bias is indicated by the intercept and slope of the regression line.

Supplementary figure S4. Example of digital TIL quantification with the HALO® image analysis platform. **A.** The residual cancer area was annotated by an experienced pathologist. **B**. At greater magnification (10x) the software was trained for tissue classification. Three classes where defined: tumor, stroma and no-tissue. Cellular segmentation was preformed and phenoptyping of CD3 (brown), CD20 (red) and other cells (blue) was done using the HALO multiplex IHC software package.

Supplementary figure S5. Scores from pathologists 1 and 2 were compared for the CD3/CD20-stained tissues from patients in the Residual Disease and the Good Response Cohort. **A.** and **D**. Forest plots show the concordance correlation coefficient (CCC) with the 95% confidence interval (CI) for inter-pathologist agreement on stromal and intra-tumoral TIL scoring. **B.** and **E.** Bland-Altman plots for stromal and intra-tumoral TIL scores is shown as a ratio of pathologist 1 to pathologist 2 (y axis) plotted versus the mean score for each sample (x axis). The mean ratio (central line) of these scores with the 95% limits of agreement are shown as horizontal lines. **C.** and **F.** Passing-Bablock regression analysis for stromal and intra-tumoral TIL scores from pathologist 1 (y axis) compared to pathologist 2 (x axis). The regression lines with the 95% CI (colored band) are represented. Constant and proportional bias are indicated by the intercept and slope of the regression line, respectively.

Supplementary figure S6. Aggregate scores from pathologists 1 and 2 were compared for the H&E- and CD3/CD20-stained tissues **A.** A forest plot shows the concordance correlation coefficient (CCC) with the 95% confidence interval (CI) for inter-pathologist agreement. **B.** Bland-Altman plots show the ratio of pathologist 1 to pathologist 2 (y axis) plotted against the geometric mean score for each sample (x axis). The mean ratio (central line) of these scores with the 95% limits of agreement are shown as horizontal lines. The same comparison was performed for patients in the Good Response Cohort **C.** A forest plot of the CCC with 95% CI for inter-pathologist agreement. **D.** Bland-Altman plots show the ratio of pathologist 1 to pathologist 2 (y axis) plotted against the mean scores for each sample (x axis).

Supplementary figure S7. Aggregate scores from H&E- and CD3/CD20-stained tissues were compared in both cohorts. **A.** A forest plot shows the concordance correlation coefficient (CCC) with 95% confidence interval (CI) for agreement between scoring on H&E- and CD3/CD20-stains **B.** Bland-Altman plots show the ratio of scores on H&E- to CD3/CD20-stained tissues (y axis) plotted against the geometric mean scores for each sample (x axis). The mean ratio (central line) of these scores with the 95% limits of agreement are shown as horizontal lines. **C.** Passing-Bablock regression plots of scores on H&E- versus CD3/CD20-stained tissues. The regression lines with the 95% CI are indicated as a colored band. The constant and proportional bias of each comparison is indicated by the intercept and

slope of the regression line. A regression line could not be drawn for aggregate scores in the Good Response Cohort due to sample size

Supplementary figure S8. Stromal TIL scores from the RCB and RCB + regression area are shown per patient. The score of the RCB area is indicated by a black star, for the RCB + regression area is indicated by an open circle.
