# Supplementary material for: Tumor-Infiltrating Lymphocyte Scoring in Neoadjuvant-Treated Breast Cancer
Source: Cancers (Basel). 2024 Aug 20;16(16):2895. doi: 10.3390/cancers16162895 (PMC11352458; doi:10.3390/cancers16162895)
Supplement: Supplementary file 1 [file cancers-16-02895-s001.zip › TIL scoing in BC after neoadjuvant treatment - Supplementary files.pptx]

## Slide 1
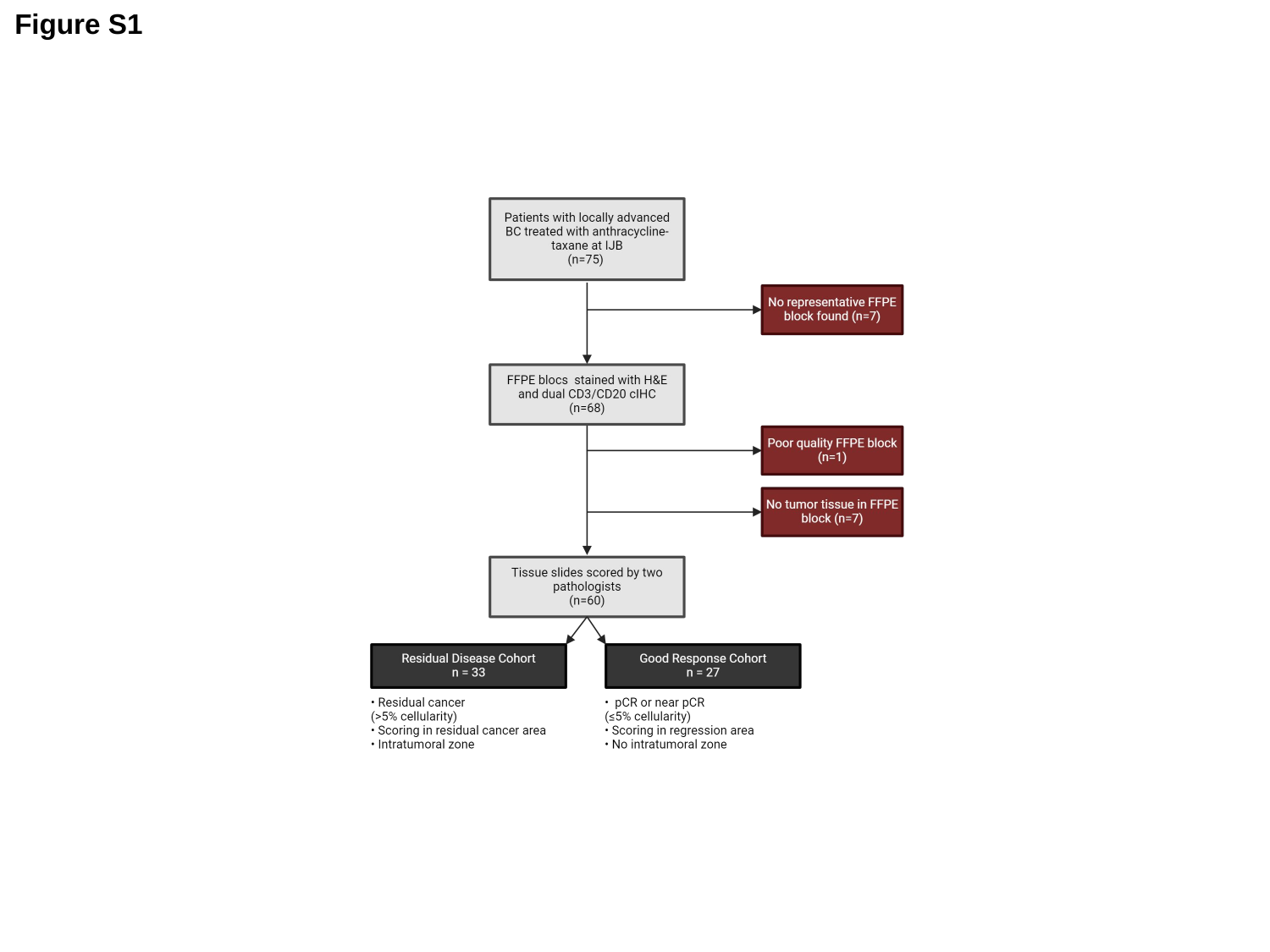

Figure S1

## Slide 2
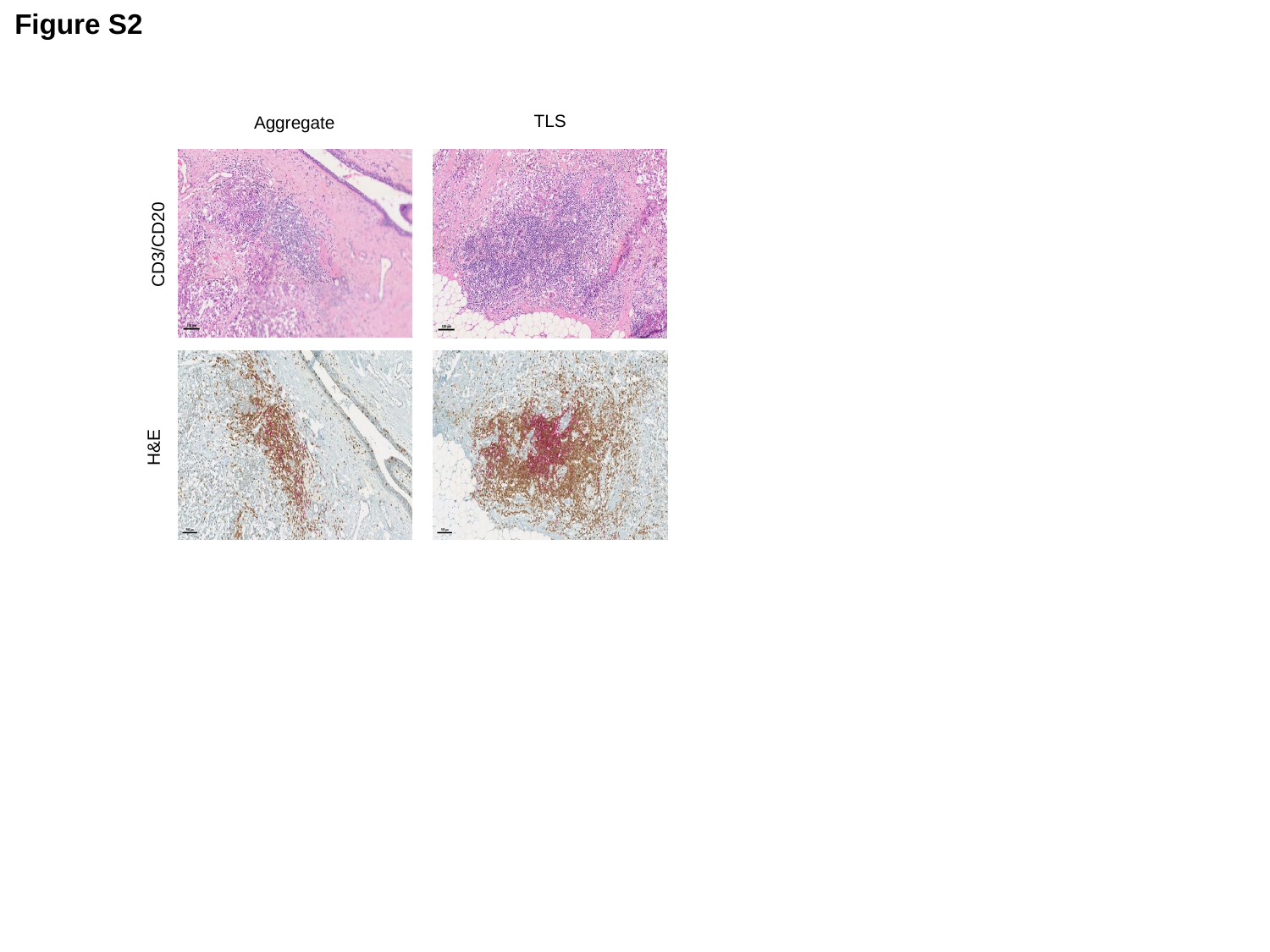

Figure S2
TLS
Aggregate
CD3/CD20
H&E

## Slide 3
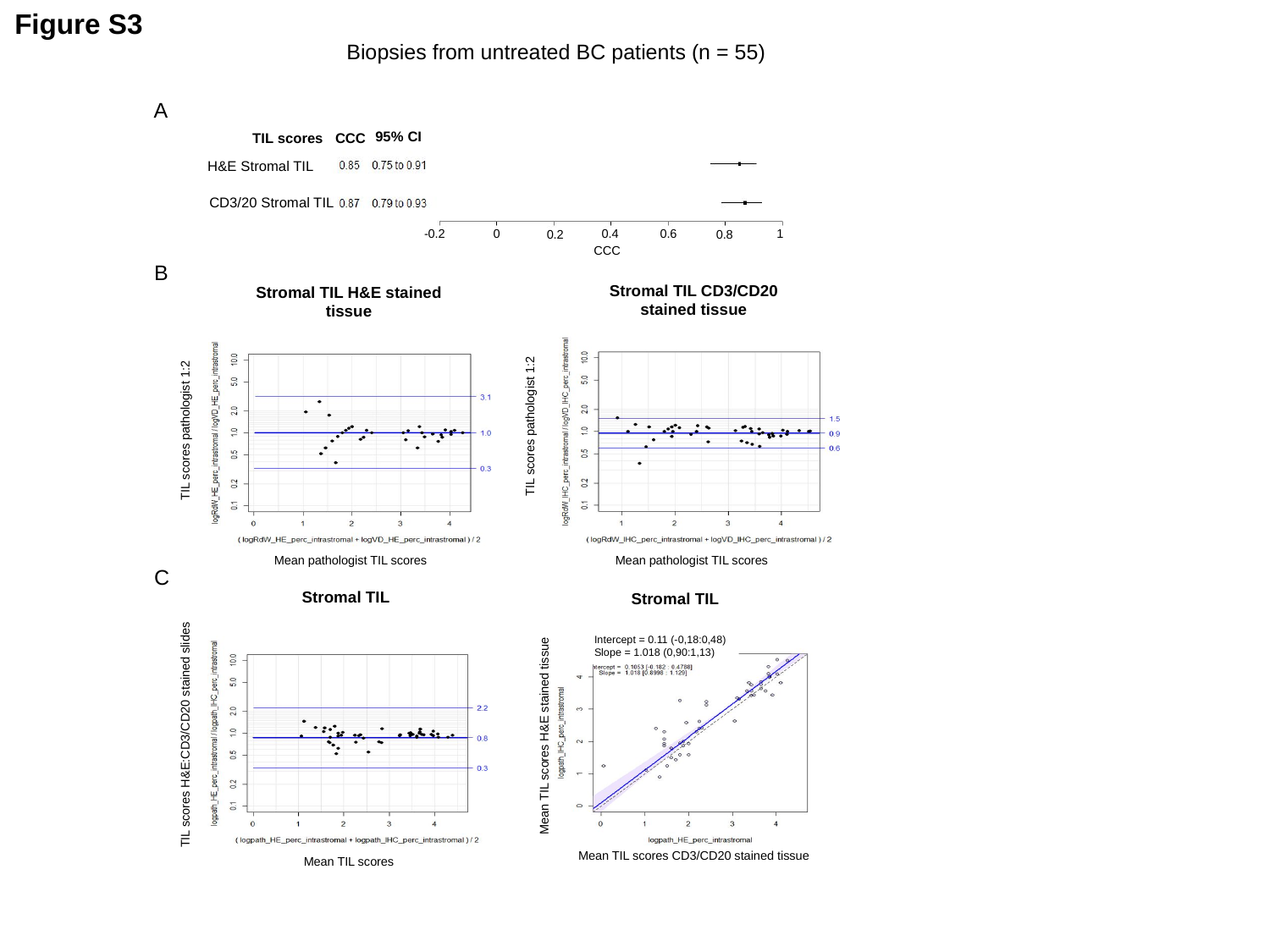

Figure S3
Biopsies from untreated BC patients (n = 55)
A
95% CI
CCC
CCC
TIL scores
H&E Stromal TIL
CD3/20 Stromal TIL
1
0.6
0
0.4
-0.2
0.8
0.2
B
Stromal TIL H&E stained tissue
TIL scores pathologist 1:2
Mean pathologist TIL scores
TIL scores pathologist 1:2
Stromal TIL CD3/CD20 stained tissue
Mean pathologist TIL scores
C
Stromal TIL
TIL scores H&E:CD3/CD20 stained slides
Mean TIL scores
Stromal TIL
Mean TIL scores H&E stained tissue
Mean TIL scores CD3/CD20 stained tissue
Intercept = 0.11 (-0,18:0,48)
Slope = 1.018 (0,90:1,13)

## Slide 4
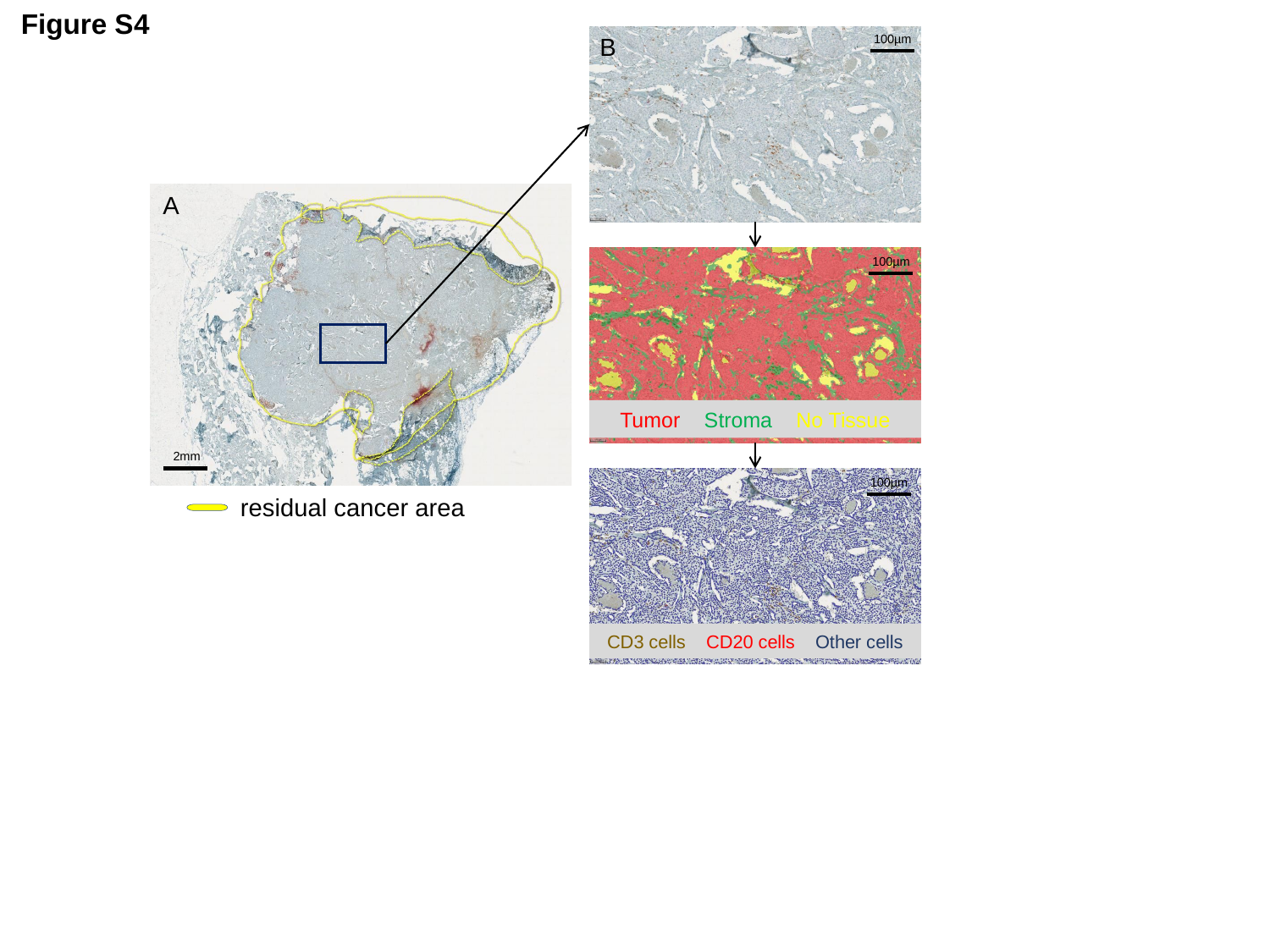

Figure S4
100µm
B
residual cancer area
A
Tumor Stroma No Tissue
CD3 cells CD20 cells Other cells
100µm
2mm
100µm

## Slide 5
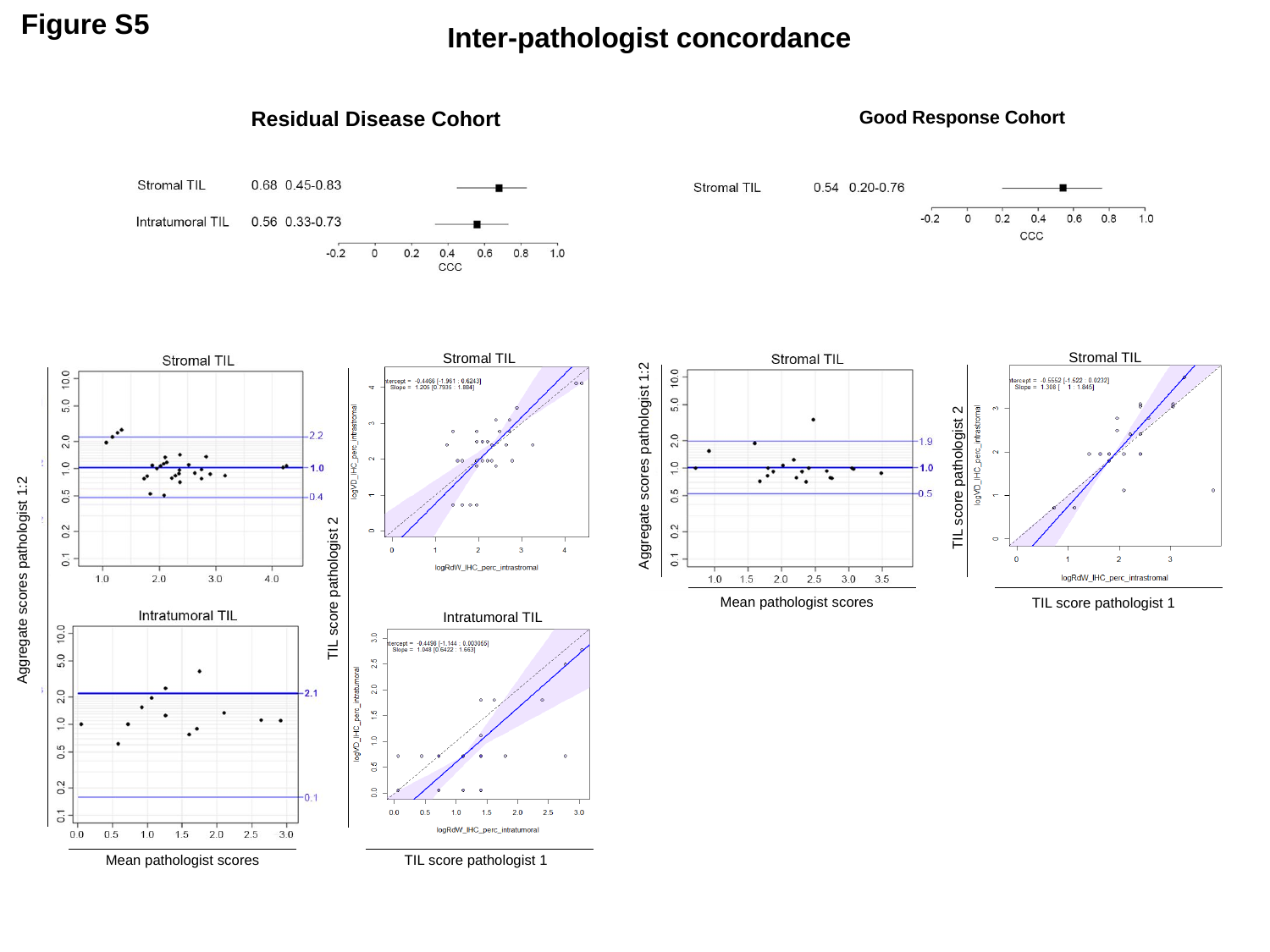

Figure S5
Inter-pathologist concordance
Residual Disease Cohort
Good Response Cohort
Stromal TIL
Stromal TIL
Aggregate scores pathologist 1:2
TIL score pathologist 2
Aggregate scores pathologist 1:2
TIL score pathologist 2
Mean pathologist scores
TIL score pathologist 1
Intratumoral TIL
Mean pathologist scores
TIL score pathologist 1

## Slide 6
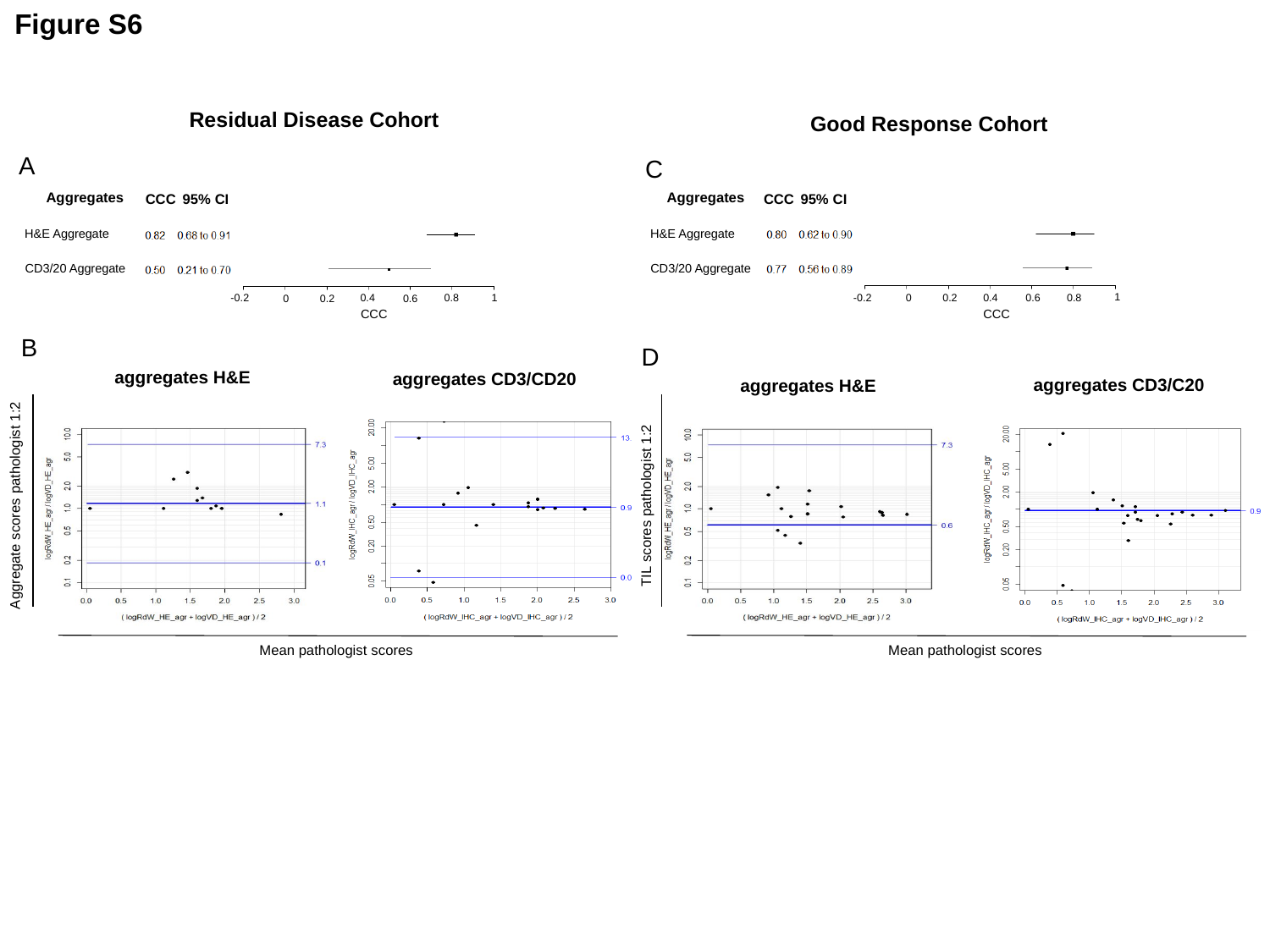

Figure S6
Residual Disease Cohort
Good Response Cohort
A
C
Aggregates
CCC
95% CI
Aggregates
CCC
95% CI
H&E Aggregate
H&E Aggregate
CD3/20 Aggregate
CD3/20 Aggregate
1
1
0.4
-0.2
0.4
0.8
-0.2
0.2
0.8
0
0.6
0.2
0
0.6
CCC
CCC
B
aggregates H&E
aggregates CD3/CD20
Aggregate scores pathologist 1:2
Mean pathologist scores
D
aggregates H&E
aggregates CD3/C20
TIL scores pathologist 1:2
Mean pathologist scores

## Slide 7
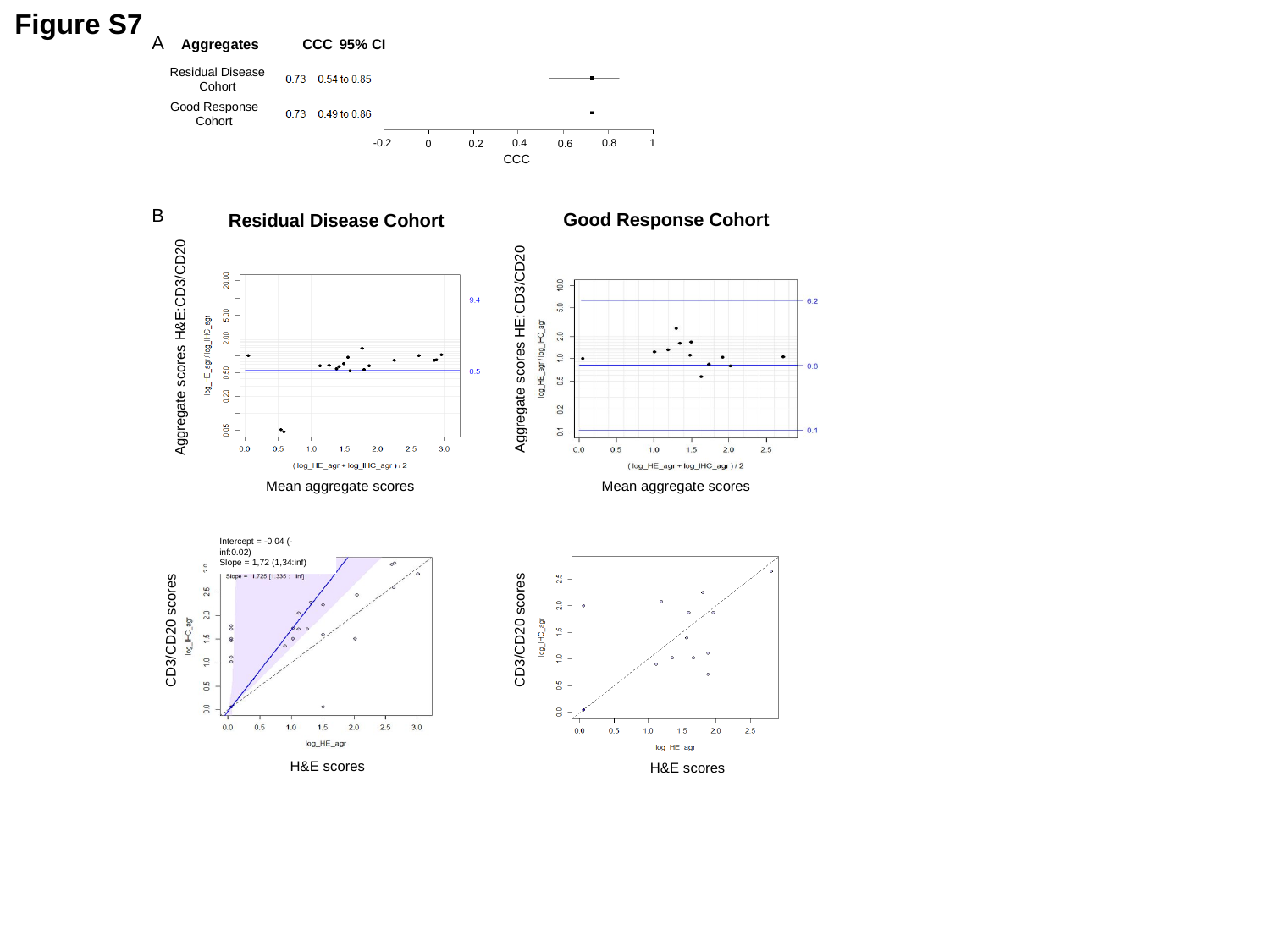

Figure S7
A
Aggregates
CCC
95% CI
B
Aggregate scores H&E:CD3/CD20
Mean aggregate scores
Good Response Cohort
Residual Disease Cohort
Aggregate scores HE:CD3/CD20
Mean aggregate scores
H&E scores
CD3/CD20 scores
Intercept = -0.04 (-inf:0.02)
Slope = 1,72 (1,34:inf)
CD3/CD20 scores
H&E scores
Residual Disease
Cohort
Good Response
Cohort
1
0.4
-0.2
0.8
0.2
0
0.6
CCC

## Slide 8
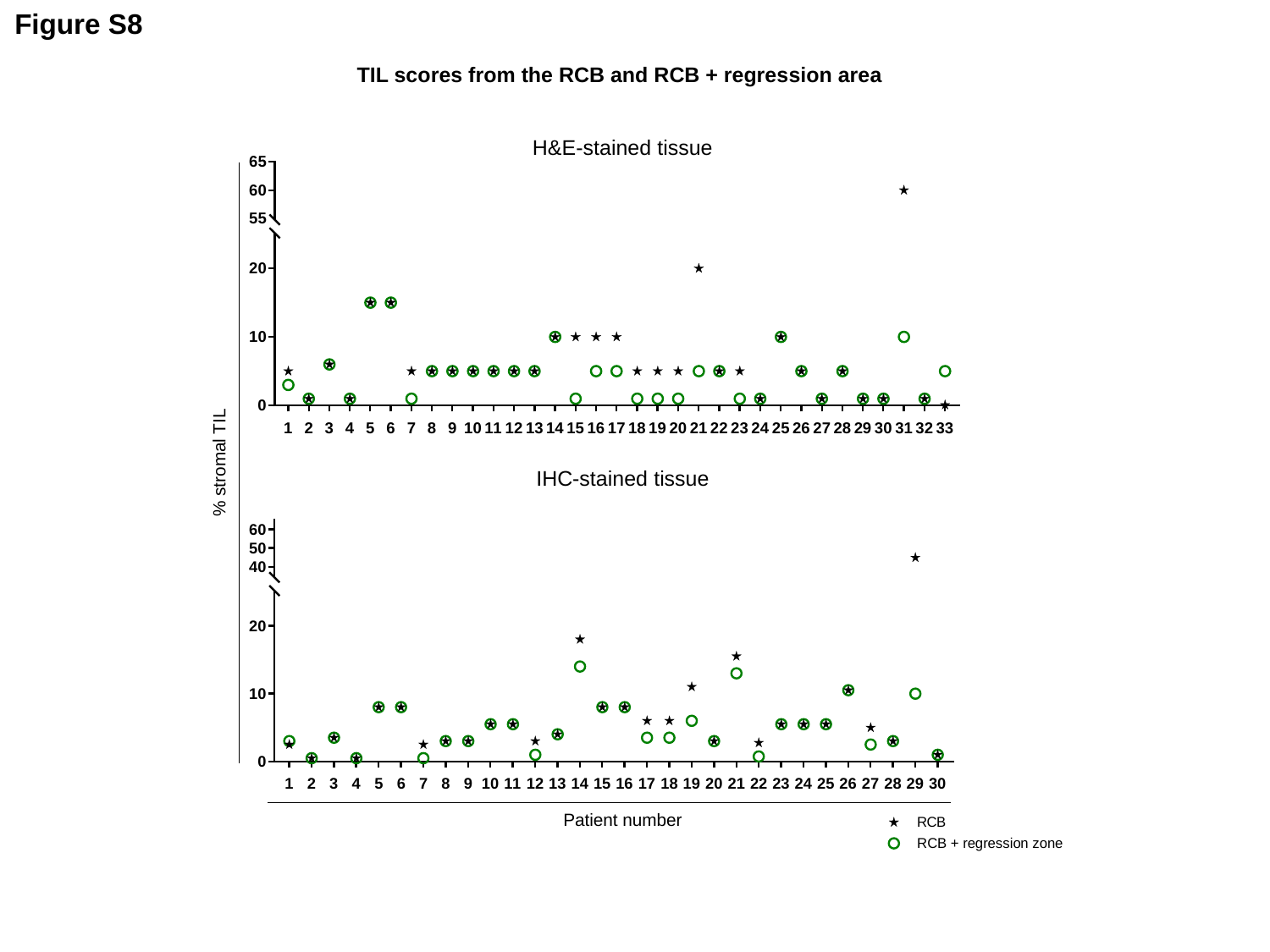

Figure S8
TIL scores from the RCB and RCB + regression area
H&E-stained tissue
% stromal TIL
IHC-stained tissue
Patient number
